# Exogenous Melatonin Suppresses Dark-Induced Leaf Senescence by Activating the Superoxide Dismutase-Catalase Antioxidant Pathway and Down-Regulating Chlorophyll Degradation in Excised Leaves of Perennial Ryegrass (*Lolium perenne* L.)

**DOI:** 10.3389/fpls.2016.01500

**Published:** 2016-10-05

**Authors:** Jing Zhang, Huibin Li, Bin Xu, Jing Li, Bingru Huang

**Affiliations:** ^1^College of Agro-grassland Science, Nanjing Agricultural UniversityNanjing, China; ^2^Department of Plant Biology and Pathology, Rutgers UniversityNew Brunswick, NJ, USA; ^3^Department of Crop Science, Agricultural University of HebeiBaoding, China; ^4^Department of Grassland Science, China Agricultural UniversityBeijing, China

**Keywords:** leaf senescence, chlorophyll degradation, reactive oxygen species, antioxidant, melatonin

## Abstract

Leaf senescence is a typical symptom in plants exposed to dark and may be regulated by plant growth regulators. The objective of this study was to determine whether exogenous application of melatonin (N-acetyl-5-methoxytryptamine) suppresses dark-induced leaf senescence and the effects of melatonin on reactive oxygen species (ROS) scavenging system and chlorophyll degradation pathway in perennial grass species. Mature perennial ryegrass (*Lolium perenne L.* cv. ‘Pinnacle’) leaves were excised and incubated in 3 mM 2-(N-morpholino) ethanesulfonic buffer (pH 5.8) supplemented with melatonin or water (control) and exposed to dark treatment for 8 days. Leaves treated with melatonin maintained significantly higher endogenous melatonin level, chlorophyll content, photochemical efficiency, and cell membrane stability expressed by lower electrolyte leakage and malondialdehyde (MDA) content compared to the control. Exogenous melatonin treatment also reduced the transcript level of chlorophyll degradation-associated genes and senescence marker genes (*LpSAG12.1*, *Lph36*, and *Lpl69*) during the dark treatment. The endogenous O_2_^-^ production rate and H_2_O_2_ content were significantly lower in these excised leaves treated with melatonin compared to the water control. Exogenous melatonin treatment caused increases in enzymatic activity and transcript levels of superoxide dismutase and catalase but had no significant effects on ascorbate peroxidase, glutathione reductase, dehydroascorbate reductase, and monohydroascorbate reductase. The content of non-enzymatic antioxidants, such as ascorbate and dehydroascorbate, were decreased by melatonin treatment, while the content of glutathione and oxidized glutathione was not affected by melatonin. These results suggest that the suppression of dark-induced leaf senescence by exogenous melatonin may be associated with its roles in regulating ROS scavenging through activating the superoxide dismutase-catalase enzymatic antioxidant pathway and down-regulating chlorophyll degradation in perennial ryegrass.

## Introduction

Leaf senescence is a highly regulated natural process during leaf development and can be accelerated by abiotic stresses such as low light conditions (Causin et al., 2009; Brouwer et al., 2012). This process is characterized by the loss of net chlorophyll content due to chlorophyll degradation (Matile et al., 1999; Hörtensteiner, 2006). Chlorophyll degradation is catalyzed by at least six chlorophyll catabolic enzymes (CCEs), such as Non-Yellow Coloring1 (NYC1), NYC1-like (NOL), Chl a reductase (HCAR), pheophytin pheophorbide hydrolyase (PPH), pheide a oxidase (PAO), and red chlorophyll catabolite reductase (RCCR) (Barry et al., 2008; Hörtensteiner, 2009). In addition, the stay-green gene (*SGR*), cloned in many plant species, has also been found to regulate chlorophyll degradation by interacting with the six CCEs (Park et al., 2007; Sakuraba et al., 2012). The transcription of these seven chlorophyll degradation-associated genes is upregulated during the senescence process (Matile et al., 1999; Park et al., 2007; Jespersen et al., 2016). Similar to chlorophyll degradation-associated genes, the transcription of senescence marker genes, such as *SAG12*, *h36*, and *l69*, is also upregulated during leaf senescence (Lee et al., 2001; Zhou et al., 2013).

Stress-induced leaf senescence, such as that induced by prolonged darkness, has also been associated with increased production of reactive oxygen species (ROS) and weakening of antioxidant scavenging systems (Wang et al., 2012). The ROS scavenging system in higher plants involves both enzymatic and non-enzymatic components. Superoxide dismutase (SOD, EC 1.15.1.1) serves as the initial defense by converting O_2_^-^ to H_2_O_2_, which is subsequently converted to H_2_O through four pathways: (1) water-water cycle converting H_2_O_2_ to H_2_O using ascorbate (AsA) as a reductant catalyzed by ascorbate peroxidase (APX, EC 1.11.1.11); (2) the ascorbate-glutathione cycle converting H_2_O_2_ to H_2_O using AsA as a reductant catalyzed by APX (EC 1.11.1.11), monodehydroascorbate reductase (MDHAR, EC 1.6.5.4), glutathione-dependent dehydroascorbate reductase (DHAR, EC 1.8.5.1), and glutathione reductase (GR, EC 1.6.4.2); (3) the glutathione peroxidase (GPX, EC 1.11.1.9) cycle detoxifying H_2_O_2_ by GPX and GR using GSH as a reductant; and (4) catalase (CAT, EC 1.11.1.6) converting H_2_O_2_ to O_2_ directly (Mittler, 2002; Blokhina et al., 2003; Ahmad et al., 2008). Metabolic factors which suppress detoxify ROS accumulation by activating any of the three enzymatic antioxidant pathways or non-enzymatic antioxidant accumulation may be effective in suppressing stress-induced leaf senescence associated with oxidative damage.

Since melatonin was first detected in edible plants in 1995, the biosynthetic pathway and physiological function of melatonin in plants have been widely studied (Dubbels et al., 1995; Hattori et al., 1995). The highest content of melatonin was observed in the night, and the amount of this molecule is reduced in the light (Murch and Saxena, 2002). Recently, a lot of studies have demonstrated that melatonin is synthesized from tryptophan in plants; this synthetic pathway is catalyzed by four enzymes including tryptophan decarboxylase (TDC, E.C. 4.1.1.28), tryptophan 5-hydroxylase (T5H, E.C. 1.14.16.4), serotonin *N*-acetyltransferase (SANT, E.C. 2.3.1.87), and hydroxyindole-*O*-methytransferase (HIOMT, E.C. 2.1.1.4) (Kang et al., 2007a,b, 2011; Byeon and Back, 2014). It has been found to exhibit plant growth regulatory functions including regulating seed germination, growth of roots and shoots, light signaling transduction, flowering, fruit ripening, and seed maturation (Kang et al., 2010; Okazaki et al., 2010; Byeon et al., 2013; Zhao et al., 2013). Additionally, melatonin serves a role in delaying plant leaf senescence induced by abiotic or biotic stresses (Wang et al., 2012, 2013a,b; Zhang et al., 2014; Shi et al., 2015b). Several independent studies suggest that melatonin exhibits ROS-scavenging properties which may mitigate stress-induced leaf senescence during drought, heat, cold, salinity, and darkness (Zang et al., 1998; Acuña-Castroviejo et al., 2001; Tan et al., 2002; Lei et al., 2004; Shi et al., 2015a). Wang et al. (2012) reported that melatonin delayed senescence of detached apple leaves; this was attributed to the regulation of the ascorbate-glutathione cycle of ROS scavenging system as demonstrated by increased activity of enzymatic antioxidant, such as APX and MDHAR, as well as the content of non-enzymatic antioxidants such as free AsA, total AsA, free GSH, and total GSH. As mentioned above, several antioxidant pathways are involved in oxidative defense, though the major antioxidant pathways regulated by melatonin and the roles of melatonin in controlling dark-induced leaf senescence by means of ROS scavenging are not completely understood.

Leaf senescence is a major problem causing the decline in forage value and turf quality of perennial grasses in heavily shaded areas in landscape, sports fields, and meadowlands. However, the regulation of melatonin on dark-induced chlorosis and the transcription of chlorophyll degradation genes have not been systematically studied, especially in perennial grass species. Investigating the mechanisms of melatonin regulating dark-induced leaf senescence of perennial grasses is important for improving turf quality of perennial grasses in shaded areas. The objectives of this study were (i) to determine physiological effects of exogenous melatonin in dark-induced leaf senescence of perennial ryegrass, (ii) to investigate the antioxidant roles of melatonin in suppressing dark-induced leaf senescence, and (iii) to determine major ROS scavenging pathways regulated by melatonin during dark-induced leaf senescence in perennial ryegrass.

## Materials and Methods

### Plant Materials and Treatments

Plants of perennial ryegrass (*Lolium perenne* cv. ‘pinnacle’) were established from seed and maintained by irrigating with half-strength Hoagland nutrient solution every 7 days (Hoagland and Arnon, 1950) in a greenhouse at Rutgers University, New Brunswick, NJ, USA. Plants were grown in plastic pots (20 cm diameter and 25 cm height) with a mix of soil and peat (3:1 v/v) and maintained in the greenhouse for 60 days and the blade of last mature leaves were detached and used for melatonin and dark treatments.

For testing the effects of melatonin on dark-induced leaf senescence of perennial ryegrass, detached leaves were treated following the protocol reported by Zhang et al. (2016). Briefly, leaves were washed with deionized water and then placed between paper towels soaked in 3 mM 2-(N-morpholino) ethanesulfonic (MES) buffer (pH 5.8). One set of leaves (60 leaves in each set) were incubated in MES buffer (control) and three sets of leaves were incubated in MES buffer solution containing 20, 50, or 100 μM melatonin. The control leaves and those treated with different concentrations of melatonin were maintained in dark for 8 days to induce leaf senescence in a growth chamber (Conviron, Winnipeg, Canada). The growth chamber was maintained at 22/17° (14 h/10 h) temperature without light to impose dark stress. The experiment was repeated three times.

### Physiological Analysis

Senescence-associated physiological parameters, including leaf chlorophyll content, photochemical efficiency (*F*v/*F*m), electrolyte leakage (EL), and malondialdehyde (MDA) content were evaluated to determine effects of exogenous melatonin on leaf senescence induced by the dark treatment. MDA is the product of membrane lipid peroxidation; therefore, the higher MDA content indicated more membrane damage.

Chlorophyll was extracted by soaking leaves in dimethyl sulfoxide (DMSO) in darkness for 72 h and then measuring for absorbance of chlorophyll extracts at 663 and 645 nm with a spectrophotometer (Spectronic Instruments, Rochester, NY, USA). Chlorophyll content was calculated using the equations described by Barnes et al. (1992). Photochemical efficiency (*F*v/*F*m) was determined using a fluorescence meter (Dynamax, Houston, TX, USA) as described by Oxborough and Baker (1997). Electrolyte leakage was measured using the methods described Murray et al. (1989). Briefly, 0.2 g leaves were washed three times and then immersed in 35 mL of deionized water. Initial conductivity (Ci) was measured with a conductivity meter (YSI Model 32, Yellow Spring, OH, USA) after shaking for 24 h. Leaves were autoclaved for 20 min and the resulting conductance (Cmax) was then measured. The electrolyte leakage was calculated as 100 × C*_i_*/C*_max_*. MDA content, another membrane stability parameter, was quantified according to the method described by Zhang and Kirkham (1996). Briefly, 0.5 g of leaf tissue was ground to powder using liquid nitrogen, and then transferred into 6 mL cold 5% trichloroacetic acid (TCA) and mixed well. The homogenate was centrifuged at 10,000 *g* for 20 min at 4°C; 1 mL of the supernatant was transferred into a 15 mL tube and mixed with 2 mL 20% TCA containing 0.5% thiobarbituric acid. The mixture was incubated at 95°Cfor 30 min, quickly cooled to room temperature in an ice-water bath, and then centrifuged at 10,000 *g* for 10 min. The absorbance of the supernatant was measured at 532 and 600 nm using a spectrophotometer (Spectronic Instruments, Rochester, NY, USA). The amount of MDA was calculated based on the extinction coefficient of 155 mM^-1^ cm^-1^ (Heath and Packer, 1968).

### Quantification of Endogenous Melatonin Content by Enzyme-Linked Immunosorbent Assay (ELISA)

Melatonin from the leaves was extracted following the manufacturer’s instruction of the Melatonin ELISA Kit (Enzo Life Sciences, Farmingdale, NY, USA). In Brief, 0.15 g of leaf samples was ground to power using liquid nitrogen, homogenized in 125 μL 1X stabilizer and then added 750 μL cold ethyl acetate and vortexed well. After incubated on ice for 5 min, spin the mix at 1000 g for 10 min. Then, the organic layer was transferred to a fresh centrifuge tube and dry to completeness. The pellet was suspended in 125–250 μL of 1X stabilizer for quantification of melatonin according to the manufacturer’s instruction of the Melatonin ELISA Kit (Enzo Life Sciences).

### Quantifications of Superoxide (O_2_^-^) and Hydrogen Peroxide (H_2_O_2_)

In order to determine whether melatonin effects on dark-induced leaf senescence was related to the alteration of ROS production, the O_2_^-^ production rate and H_2_O_2_ content were evaluated in leaves treated with or without melatonin. The O_2_^-^ production rate was measured according to methods described by Elstner and Heupel (1976). Briefly, 0.1 g leaf tissue was ground to power using liquid nitrogen, homogenized in 3 mL 65 mM potassium phosphate buffer (PBS) (pH 7.8), and centrifuged at 10000 *g* for 15 min at 4°C. Simultaneously, 0.5 mL PBS (pH 7.8) and 0.1 mL 10 mM hydroxylamine hydrochloride were mixed and incubated at 25°C for 10 min. Then, 0.5 mL supernatant was added to the PBS and hydroxylamine hydrochloride mixture and incubated at 25°C for 20 min. Following incubation, 1 mL 58 mM sulfonamides and 1 mL 7 mM naphthylamine were added to the mixture, respectively, and incubated at 25°C for another 20 min. Three mL of chloroform was added to the reaction mixture and vortexed, followed by centrifugation at 10000 *g* for 3 min. The absorbance of the upper phase was measured at 530 nm using a spectrophotometer (Spectronic Instruments, Rochester, NY, USA). The production rate was calculated according to the formula described by Elstner and Heupel (1976).

The content of H_2_O_2_ was measured according to the method described by Velikova et al. (2000). Briefly, 0.5 g leaf tissues was ground to powder using liquid nitrogen and homogenized in 5 mL cold 0.1% (w/v) TCA. The homogenate was centrifuged at 12000 *g* for 15 min and 0.5 mL of the supernatant was added to 0.5 mL 10 mM potassium phosphate buffer (pH 7.0) and 1 mL 1 M KI. The mixture was incubated in dark at 28°C for 15 min. The absorbance was measured at 390 nm. The content of H_2_O_2_ was based on a standard curve generated with known H_2_O_2_ concentrations.

Histochemical staining for O_2_^-^ and H_2_O_2_ was also performed for visual assessment of ROS production. Staining of O_2_^-^ was performed following the protocol described by Dunand et al. (2007). Leaves were stained with 2 mM nitro blue tetrazolium (NBT) in 20 mM phosphate-buffered saline (PBS; pH 6.8) for 8 h and subsequently decolorized by boiling in ethanol. Staining of H_2_O_2_ was performed following the protocol described as Thordal-Christensen et al. (1997). Leaves were incubated in 1% (w/v) 3-diaminobenzinidine (DAB; pH 3.8) for 16 h and subsequently decolorized by boiling in ethanol.

### Analysis of Non-enzymatic Antioxidant Content and Enzyme Activities

In order to determine whether melatonin effects on dark-induced leaf senescence was related to changes in antioxidant metabolism, endogenous content of non-enzymatic antioxidants (AsA, DHA, GSH, and GSSG) and activities of antioxidant enzymes were measured in excised leaves treated with or without melatonin. Endogenous contents of AsA, DHA, GSH, and GSSG were quantified according to the method described by Griffith (1980) and Ma et al. (2008), with slight modifications. In brief, 0.5 g of leaf tissue was ground to powder using liquid nitrogen; 6 mL cold 5% TCA was added and the mixture was mixed. The homogenate was centrifuged at 10,000 *g* for 20 min at 4°C and the supernatant was saved for further analysis.

For total AsA content analysis, 0.8 mL supernatant was incubated in 200 mM PBS (pH 7.4) and 1.5 mM dithiothreitol (DTT) mixture at room temperature for 50 min. After incubation, 200 μL 0.5% (w/v) N-ethylmaleimide (NEM) was added to remove excess DTT. Then 1 mL 10% (w/v) TCA, 800 μL 42% (w/v) o-phosphoric acid, 800 μL 65 mM 2,2′-dipyridyl in 70% (v/v) ethanol, and 400 μL 3% (w/v) FeCl_3_ were added to the reaction mixture. The reaction was incubated at 42°C for 1 h in a water bath and the absorbance was quantified at 525 nm. Free AsA was analyzed using the similar method described above except DTT and NEM were substituted with 400 μL deionized water. Total and free AsA contents were determined based on a standard curve generated with known AsA concentrations. DHA was estimated from the difference between total AsA and free AsA.

For total glutathione content analysis, 0.5 mL supernatant was mixed with 1.0 mL 0.2 M PBS (pH 7.0), 0.1 mL 0.5% (w/v) dithiobis-2-nitrobenzoic acid (DTNB, dissolved in DMSO), 0.2 mL 2 mM NADPH, and 0.2 mL 50 mM ethylenediaminetetraacetic acid (EDTA). The reaction was initiated by adding three units of GR and the absorbance was quantified at 412 nm for 1 min. GSSG was analyzed in a same method as above, except for that the 1 mL supernatant was first incubated with 50 μL 2-vinylpyridine at room temperature for 1 h to derivatize GSH. Total glutathione and GSSG contents were determined based on a standard curve generated with known GSH concentrations. GSH was estimated from the difference between total glutathione and GSSG.

For the analysis of enzymatic antioxidant activity, crude enzyme solution was extracted as described by Zhang and Kirkham (1996). Briefly, 0.3 g leaf tissue was ground to powder using liquid nitrogen and then homogenized in 3 mL cold 50 mM PBS (pH 7.8) containing 1% (w/v) polyvinylpyrrolidone (PVP) and 0.2 mM EDTA. Homogenates were then centrifuged at 15,000 *g* at 4°C for 20 min and the supernatant was saved for enzyme activity analysis. SOD activity was determined by measuring the absorbance at 560 nm and is defined as the amount of enzyme required to cause 50% inhibition of the rate of nitroblue tetrazolium chloride reduction (Meloni et al., 2003). CAT activity was determined by measuring the decline in absorbance at 240 nm as H_2_O_2_ concentration reduces (Chance and Maehly, 1955). APX activity was determined by measuring the decrease in absorbance at 290 nm for 1 min (Nakano and Asada, 1981). GR activity was measured by measuring the decrease in absorbance at 340 nm for 4 min (Cakmak et al., 1993). DHAR activity was measured by measuring the decrease in absorbance at 265 nm for 1 min (Nakano and Asada, 1981). MDHAR activity was determined by measuring the change in absorbance at 340 nm for 24 s (Cakmak et al., 1993). GPX activity was assayed by measuring the change in absorbance at 340 nm as NADPH oxidation occurred (Klapheck et al., 1990). Protein content of crude enzyme solution was determined as described by Bradford (1976). One unit enzyme activity was defined as the absorbance change per minute.

### Analysis of Gene Expression with Real-Time RT-PCR

In order to determine whether exogenous melatonin regulates dark-induced leaf senescence was related to the expression of senescence-related genes and antioxidant-enzyme genes, the transcript levels of six chlorophyll degradation-associated genes (*LpSGR*, *LpNYC1*, *LpNOL*, *LpPPH*, *LpPAO*, and *LpRCCR1*) and three senescence marker genes (*LpSAG12.1*, *Lph36*, and *Lpl69*), as well as *LpMnSOD*, *LpFeSOD*, *LpCuZnSOD*, *LpCuZnSOD*, *LpCAT*, *LpAPX2*, *LpDHAR2*, *LpGR*, *LpGPX1*, *LpGPX2*, *LpGPX3*, *LpMDHAR1*, and *LpMDHAR2* were quantified in leaves treated with or without melatonin. Total RNA was extracted from leaf tissue using TRIzol reagent (Life Technologies, Grand Island, NY, USA). TURB DNA-free^TM^ reagent (Life Technologies) was used to remove contaminating genomic DNA from RNA solution. Following that, 2 μg total RNA was reverse transcribed into cDNA using the High Capacity cDNA Reverse Transcription Kit (Life Technologies, Grand Island, NY, USA). For gene expression analysis, the StepOnePlus Real-Time PCR System (Life Technologies, Grand Island, NY, USA) was used and the PCR reaction was performed with a power SYBR^^®^^ Green PCR Master mix (Applied Biosystems, Foster City, CA, USA). Primers used for qRT-PCR are listed in **Table 1** and *eIHF4A* was used as internal control (Huang et al., 2014). All reactions were performed with two technical and four biological replicates.

**Table 1:** Primers used in this study.

Gene	Accession number	Forward primer sequence (5′–3′)	Reverse primer sequence (5′–3′)
*LpSGR*	KX686494	GAGGAGGCGAACTCGAAG	GGTTGTACCACCCTTGCAG
*LpNOL*	KX686493	GCTGGCAAAGAAGTTTCTCA	ATGCTGCTCTCCAAATTCCT
*LpNYCl*	KX686491	GATCGTCTCCCAGAAGTGCT	GCCAGTCTCCTGCTTGAAC
*LpPPH*	KT345726	ACCCAGGTGATTCAGGAAAG	CCTGACCTCACCAACCTTCT
*LpPAO*	KX686495	TCAAGGCCAAGAGAAGGTCT	TGTGTGGGTGTGAATGTGAG
*LpRCCRl*	KX686492	ATGGTGCAATCGACATCACT	GAGCAGGTCCAGAATGACAA
*LpSAG12.1*	GR521200	ACTGCGACACGACAGACAAC	TGTACTCGAAGGCATTGTCC
*Lph36*	GR522352	GACCGCCCTTTGAACATAGT	CTTCAATCACGTCAGGATGG
*Lpl69*	GR514637	TCATGGACAACATAGCAGCA	TACTTTCACGCCGTCGATAC
*LpMnSOD*	GAYX01035468.1	GGGTGCTGCTTTACAAGGAT	GTCACCAGAGGGTCCTGATT
*LpFeSOD*	GAYX01012456.1	CTCCAAGGTCGTGTCCTTCT	CGACCCTCTTGCTCATGTAA
*LpCuZnSOD*	GR520345	TTTCATCACAACCCTCCTGA	AATGATGGTTGCAATTGTGG
*LpCAT*	GR519923	CACCTTCGACAAGAAGACGA	CTCGAGCAGGTGGTAGTCCT
*LpAPX2*	GR524197	CCCTCGTGGAGAAATATGCT	TCAGATAGCCTGAGGTGTGC
*LpDHAR2*	GR514004	GACTGTCCCTTCTCCCAGAG	AGAAACCACTTGGGCTTGTT
*LpGR*	GR522386	CAGCTGCTGTATTCTCCCAA	CCAGAAAGAGTGGCCCTAAG
*LpGPXl*	GR522096	AGCTGCTTGGGAGCTCTTAG	GCATCATCTGAAATGTTGGG
*LpGPX2*	GAYX01040858.1	CGAGAAGGACCTCAAGAAGC	GGTAACTGAGCACAACATTGC
*LpGPX3*	GT089062	AAGCTGTTGGAGGTTTGAGG	ACCACAGACCGGAACTTCTC
*LpMDHARl*	GR515060	AGCTCTCTGATTTCGGCACT	CAAGGCCAATGTAACCTCCT
*LpMDHAR2*	GR510203	CTAAGGTCGCTAGGGCTCAG	ACTAGGGAGCGTGGACAGAC
*LpelF4A*	G0924770	AACTCAACTTGAAGTGTTGGAGTG	AGATCTGGTCCTGGAAAGAATATG

### Statistical Analysis

Data was analyzed using two-way ANOVA for the analysis of dark treatment and melatonin effects. The differences between treatments were compared by student’s *t*-test at the probability of 0.05 and 0.01 using a statistical program (Version 12, SPSS Inc., Chicago, IL, USA). The data in all figures was expressed as means ± standard error.

## Results

### Exogenous Application of Melatonin Alleviated Dark-Induced Leaf Senescence and Increased Its Endogenous Level

**FIGURE 1:**
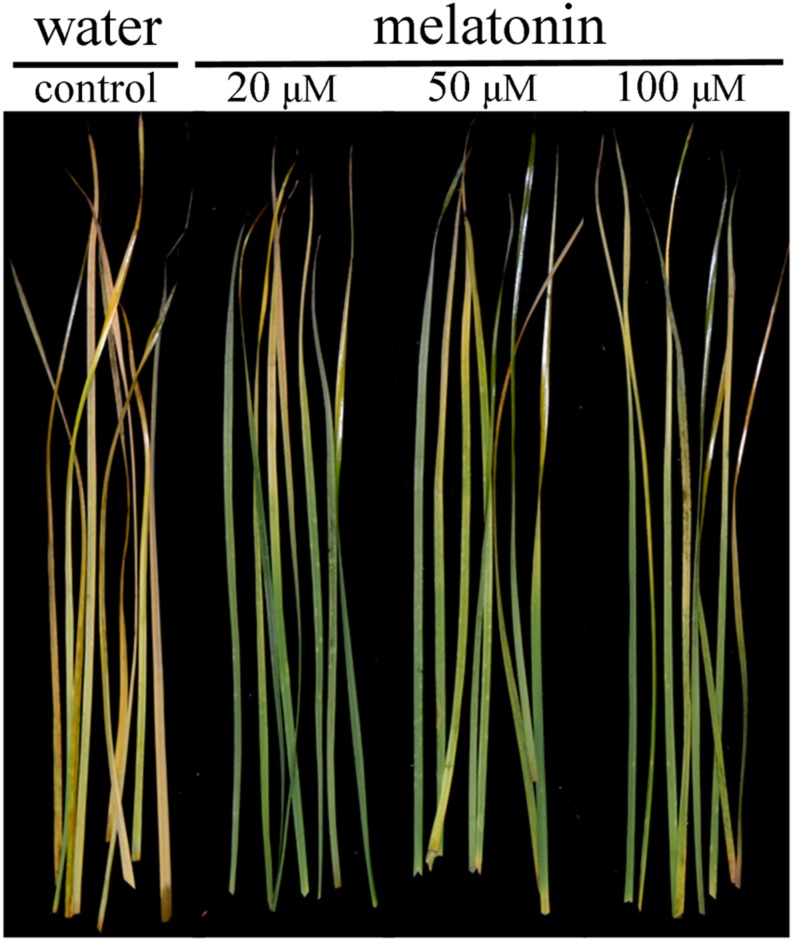
**Effects of melatonin on phenotype traits of detached perennial ryegrass leaves at day 8 after dark treatment**.

**FIGURE 2:**
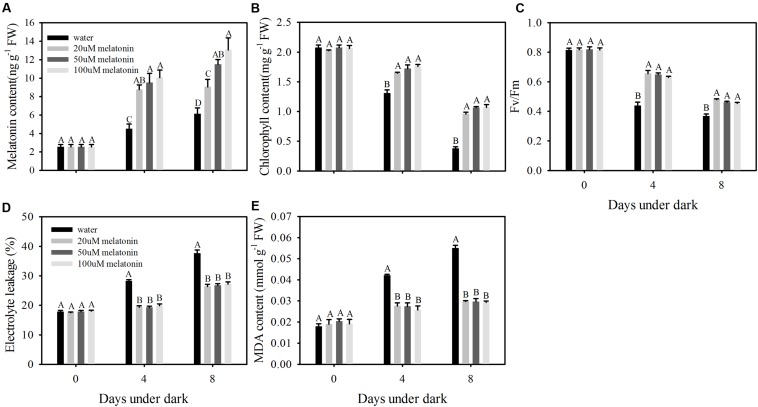
**Effects of 20, 50, and 100 μM of melatonin on endogenous melatonin level and physiological parameters of detached perennial ryegrass leaves during dark-induced senescence. (A)** Endogenous melatonin content. **(B)** Chlorophyll content. **(C)** Photochemical efficiency (*F*v/*F*m). **(D)** Electrolyte leakage. **(E)** MDA content. Different letter indicates significant differences at given day under darkness (*p* ≤ 0.05). Data are show as means ± SE (*n* = 4 in **A,B,D**,**E**; *n* = 16 in **C**).

Leaves treated with melatonin maintained greener visual color compared with the control at 8 days of dark treatment (**Figure 1**). Dark treatment increased endogenous melatonin level, and 20, 50, and 100 μM melatonin treated leaves had significantly higher level than water control (**Figure 2A**). The dark treatment significant increased the decline of chlorophyll content and photochemical efficiency in control leaves (**Figures 2B,C**). However, chlorophyll content and photochemical efficiency were significantly greater in melatonin-treated leaves than those of the control (**Figures 2B,C**). Melatonin treatment resulted in significantly lower MDA content and electrolyte leakage than the control (**Figures 2D,E**). Physiological effects of melatonin did not differ significantly among the three concentrations (20, 50, and 100 μM) (**Figures 1** and **2**). Therefore, biochemical and transcript effects of melatonin were performed for leaves treated with 20 μM, as described in the following results.

### Exogenous Melatonin Suppressing the Up-Regulation of Chlorophyll Degradation Associated Genes and Senescence Marker Genes by Dark Treatment

**FIGURE 3:**
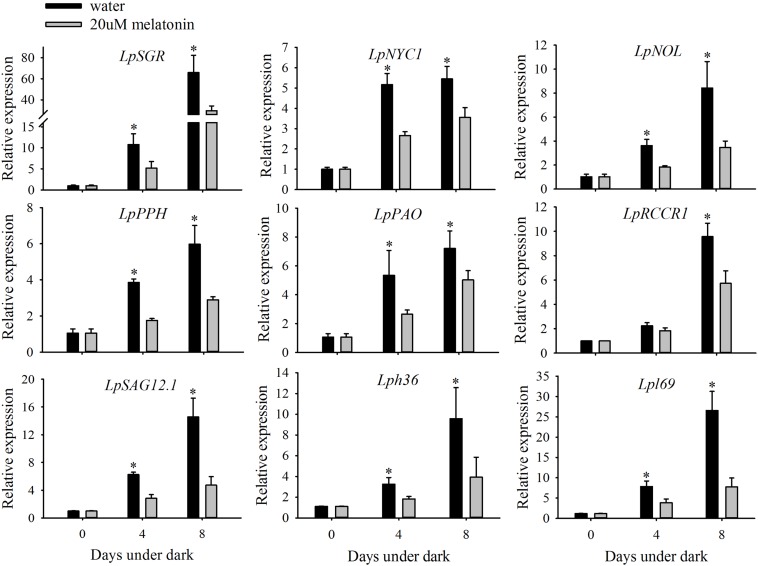
**Effects of 20 μM of melatonin on the relative expression of chlorophyll degradation genes and senescence marker genes of detached perennial ryegrass leaves during dark-induced senescence.** Chlorophyll degradation genes: *LpSGR*, *LpNYC1*, *LpNOL*, *LpPPH*, *LpPAO*, and *LpRCCR1*. Senescence marker genes: *LpSAG12.1*, *Lph36*, and *Lpl69*. Data are show as means ± SE (*n* = 4). ^∗^*P* ≤ 0.05.

The transcript changes of six chlorophyll degradation-associated genes (*LpSGR*, *LpNYC1*, *LpNOL*, *LpPPH*, *LpPAO*, and *LpRCCR1*,) and three senescence marker genes (*LpSAG12.1*, *Lph36*, and *Lpl69*) were compared between leaves treated with or without melatonin under dark treatment. The relative expression levels of all the chlorophyll degradation-associated genes and senescence marker genes were significantly increased during 4 and 8 days of dark treatment in the control (**Figure 3**). For *LpSGR*, its expression was more activated than other chlorophyll degradation genes, with 10.7- and 65.3-fold increases at 4 and 8 days of dark treatment (**Figure 3**). In addition, the relative expression level of other chlorophyll degradation-associated genes was also increased by dark treatment. However, melatonin treatment significantly suppressed the transcription of those genes compared to water control after 8 days dark treatment, with a 54.9% decrease for *LpSGR*, 34.8% decrease for *LpNYC1*, 58.8% decrease for *LpNOL*, 51.6% decrease for *LpPPH*, 30.3% decrease for *LpPAO*, and 39.7% decrease for *LpRCCR1*(**Figure 3**). The transcript patterns of the three senescence marker genes (*LpSAG12.1*, *Lph36*, and *Lpl69*) were the same as chlorophyll degradation genes.

### Exogenous Melatonin Enhances ROS Scavenging against Dark-Induced Oxidative Stress by Increasing the Activity of SOD and CAT

**FIGURE 4:**
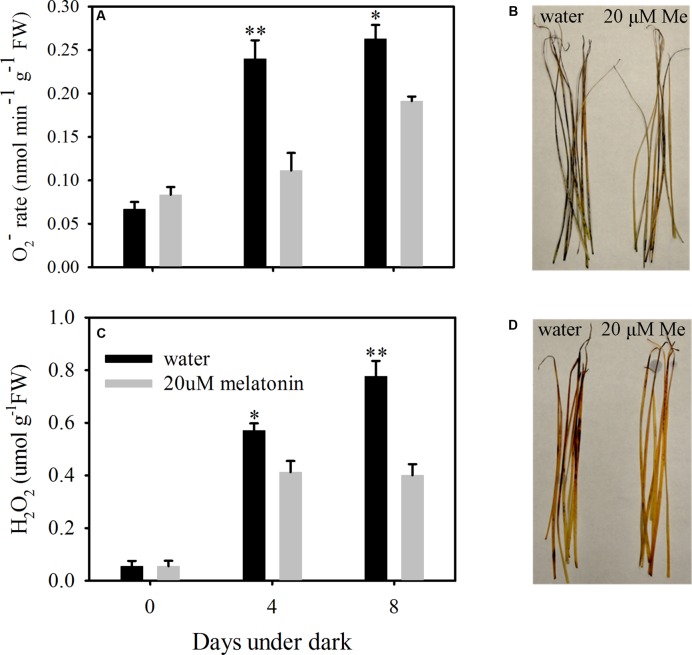
**Effects of melatonin on reactive oxygen species (ROS) in detached leaves of perennial ryegrass treated with water control and melatonin during dark-induced senescence. (A)** The production rate of super oxide (O_2_^-^). **(B)** Histochemical staining of leaves for visual localization of super oxide (O_2_^-^) at days 8 after dark treatment. **(C)** The content of hydrogen peroxide (H_2_O_2_). **(D)** Histochemical staining of leaves for visual localization of hydrogen peroxide (H_2_O_2_) at days 8 after dark treatment. Data are show as means ± SE (*n* = 4 in **A,C**). ^∗^*P* ≤ 0.05, ^∗∗^*P* ≤ 0.01.

To evaluate the dark stress induced oxidative damage and regulatory roles of melatonin on ROS scavenging, the production rate of O_2_^-^ and the H_2_O_2_ content were examined using quantitative measurement and histochemical staining. Dark treatment increased the production of O_2_^-^ of leaves without melatonin treatment by 3.19- and 3.49-fold and the H_2_O_2_ content by 9.45- and 12.86- fold at 4- and 8 days, respectively (**Figures 4A,C**). The histochemical staining also showed increased production for both O_2_^-^ and H_2_O_2_ at 8 days of dark treatment (**Figures 4B,D**). Compared to control, melatonin treatment significantly suppressed the production rate of O_2_^-^ at 4 and 8 days of dark treatment, with a 2.14- and 1.37-fold decease, respectively (**Figure 4A**). The H_2_O_2_ content was also significantly suppressed by melatonin treatment, with a 1.94- and 1.38-fold decrease at 4 and 8 days of dark treatment, respectively (**Figure 4C**). In addition, the histochemical staining also showed decreased presence for both O_2_^-^ and H_2_O_2_ with the melatonin treatment compared with the control at 8 days of dark treatment (**Figures 4B,D**).

**FIGURE 5:**
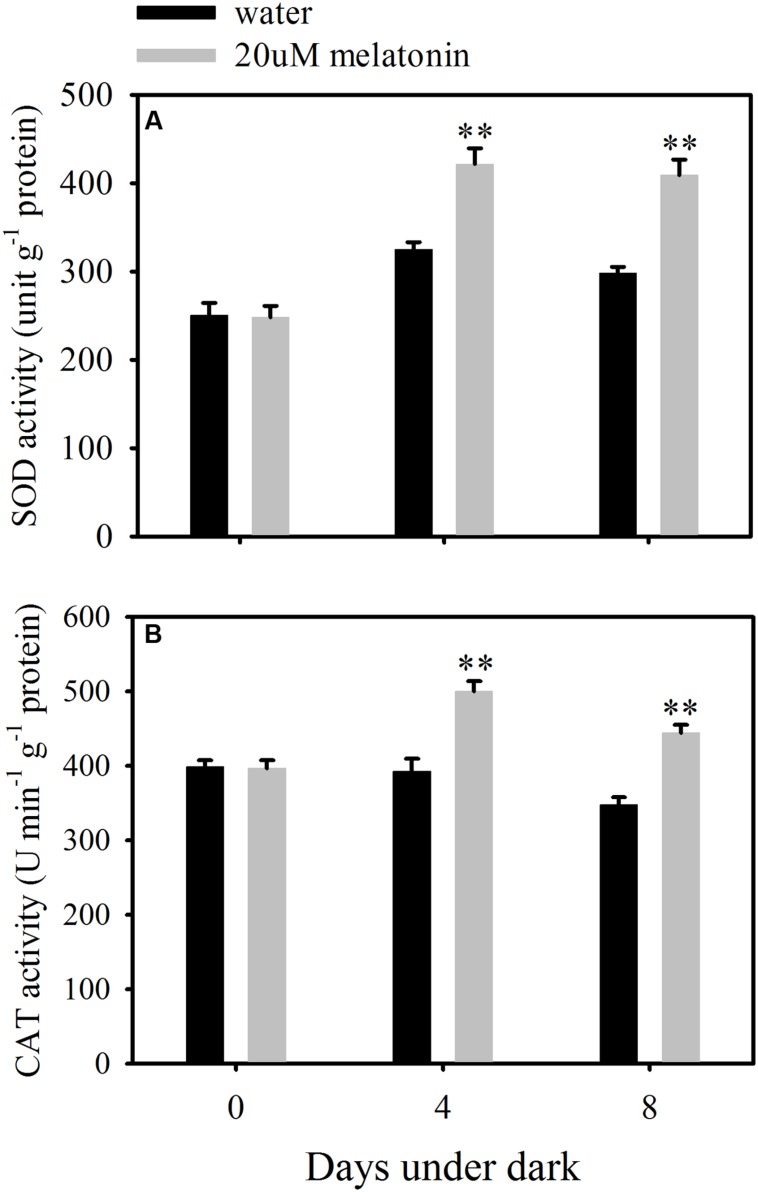
**Effects of melatonin on enzymatic activity of superoxide dismutase (SOD) (A) and catalase (CAT) (B) in detached leaves of perennial ryegrass treated with water control and melatonin during dark-induced senescence.** Data are show as means ± SE (*n* = 4). ^∗∗^*P* ≤ 0.01.

**FIGURE 6:**
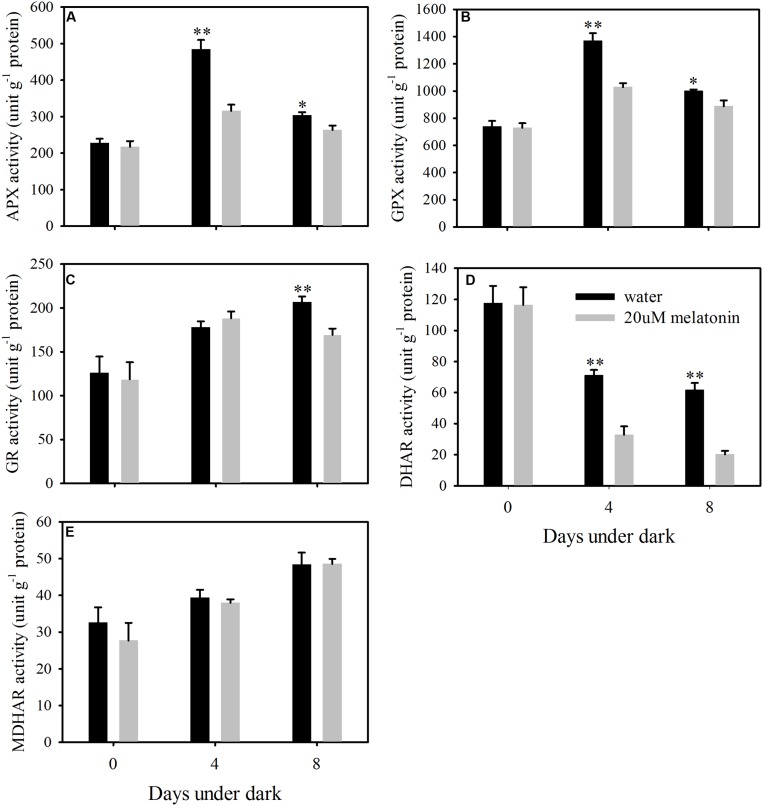
**Effects of melatonin on enzymatic activity of ascorbate peroxidase (APX) (A), glutathione peroxidase (GPX) (B), glutathione reductase (GR) (C), dehydroascorbate reductase (DHAR) (D), and monodehydroascorbate reductase (MDHAR) (E) in detached leaves of perennial ryegrass treated with water control and melatonin during dark-induced senescence.** Data are show as means ± SE (*n* = 4). ^∗^*P* ≤ 0.05, ^∗∗^*P* ≤ 0.01.

In order to evaluate whether the reduction of ROS accumulation by melatonin was related to the activation of enzymatic ROS scavenging system, the ROS scavenging enzymes and their relative expression levels were examined. Melatonin treatment significantly increased the activity of SOD and CAT, but resulted in a reduction in APX, GPX, and DHAR activity at both 4 and 8 days of dark treatment (**Figures 5** and **6A,B,D**). Melatonin had no significant effects on GR activity at 4 days of dark treatment, but reduced GR activity at 8 days of dark treatment (**Figure 6C**). For MDHAR activity, melatonin had no effect on it at either 4 or 8 days of dark treatment (**Figure 6E**).

### Exogenous Melatonin Enhances the Relative Transcription of SOD and CAT

**FIGURE 7:**
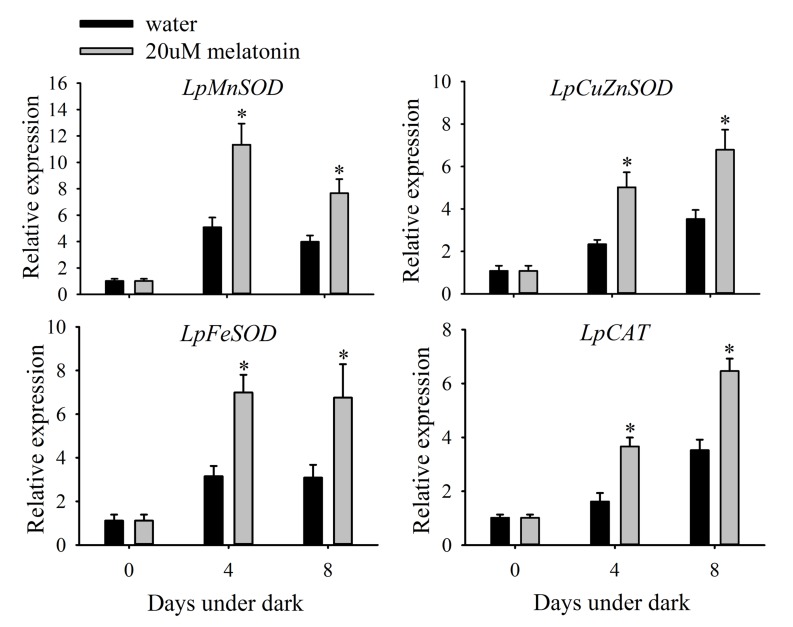
**Effects of melatonin on the relative expression of *LpMnSOD*, *LpCuZnSOD*, *LpFeSOD*, and *LpCAT* of detached perennial ryegrass leaves during dark-induced senescence.** Data are show as means ± SE (*n* = 4). ^∗^*P* ≤ 0.05.

**FIGURE 8:**
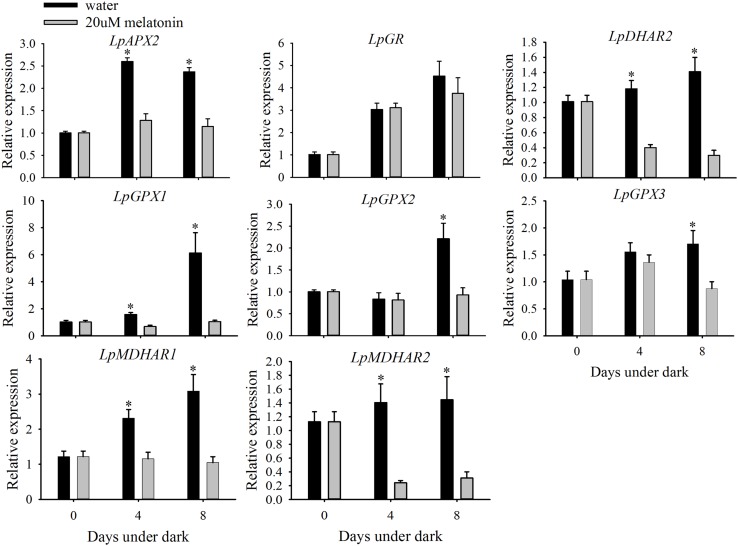
**Effects of melatonin on the relative expression of *LpAPX2*, *LpDHAR2*, *LpGR*, *LpGPX1*, *LpGPX2*, *LpGPX3*, *LpMDHAR1*, and *MDHAR2* of detached perennial ryegrass leaves during dark-induced senescence.** Data are show as means ± SE (*n* = 4). ^∗^*P* ≤ 0.05.

Leaves treated with melatonin had significantly higher relative expression levels of *LpMnSOD*, *LpFeSOD*, *LpCuZnSOD*, and *LpCuZnSOD* than the control at either 4 or 8 days of dark treatment (**Figure 7**). The relative expression level of *LpAPX2* was significantly suppressed by melatonin treatment at either 4 or 8 days of dark treatment (**Figure 8**). The expression level of *LpDHAR2* was significantly reduced by melatonin at either 4 or 8 days of dark treatment (**Figure 8**). The expression level of *LpGR* was not affected by melatonin (**Figure 8**). Melatonin significantly reduced the transcript level of *LpGPX1* at either 4 or 8 days of dark treatment (**Figure 8**). For *LpGPX2* and *LpGPX3*, melatonin significantly reduced the transcript level at 8 days of dark treatment (**Figure 8**). The expression levels of *LpMDHAR1* and *LpMDHAR2* were inhibited by melatonin.

### Melatonin Has No Beneficial Effects on Non-enzymatic Antioxidants

**FIGURE 9:**
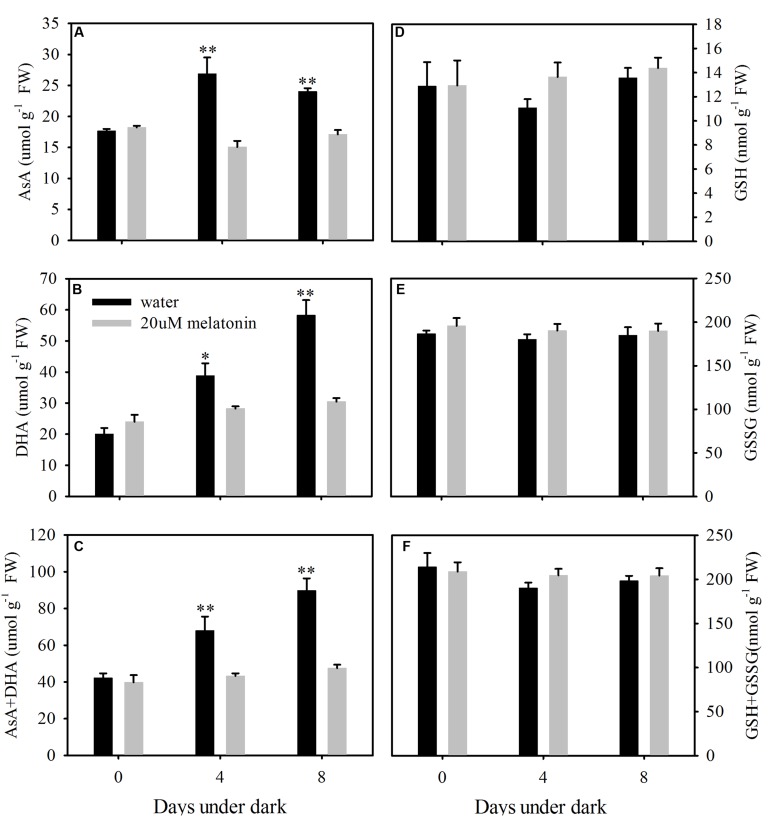
**Effects of melatonin on endogenous ascorbate (AsA) content (A), dehydroascorbate (DHA) content (B), AsA+DHA (C), glutathione (GSH) content (D), oxidized glutathione (GSSG) content (E), GSH+GSSG (F) in detached leaves of perennial ryegrass treated with water control and melatonin during dark-induced senescence.** Data are show as means ± SE (*n* = 4). ^∗^*P* ≤ 0.05, ^∗∗^*P* ≤ 0.01.

The content of free AsA, DHA, and total AsA level was significantly lower in melatonin-treated plants than those in the control at 8 days of dark treatment (**Figures 9A–C**). The AsA contents were increased by the dark treatment and this increase was suppressed by the melatonin treatment (**Figure 9A**). The endogenous free GSH, GSSG, and total GSH levels were not affected by melatonin treatment at 4 or 8 days of dark treatment (**Figures 9D–F**).

## Discussion

Physiological analysis demonstrated that exogenous treatment of excised leaves with melatonin effectively suppressed dark-induced leaf senescence, as manifested by maintaining greater chlorophyll content and photochemical efficiency, and lower MDA content and EL in leaves of perennial ryegrass exposed to the dark treatment. Previous studies with other plant species have also found the positive effects of melatonin dark-induced leaf senescence; for perennial ryegrass, the effective concentration was between 20 and 100 μM in this study. Others found 10 μM melatonin was effective for suppressing leaf senescence in barley and 10 mM for apple leaves (Arnao and Hernández-Ruiz, 2009; Wang et al., 2012). It is evident that leaves of grass or monocot species are more sensitive to melatonin than leaves of woody or dicots plants. Unlike apple, perennial ryegrass and barley are fructan accumulating species. Recently, several studies suggested that fructans could function as ROS scavenger (Keunen et al., 2013; Peshev et al., 2013). Thus, the difference of melatonin sensitivity between species could be due to the ability to synthesis fructans. In addition, exogenous application of melatonin significantly increased the endogenous melatonin level compared to water control. This may due to the absorption of exogenous melatonin or exogenous application of melatonin induced the expression of melatonin biosynthesis genes in detached perennial ryegrass leaves under dark condition. The increased endogenous melatonin may directly suppress dark-induced leaf senescence of perennial ryegrass.

Leaf senescence is typically characterized by the decline in chlorophyll content, which has been associated with the up-regulation of chlorophyll degradation-associated genes during natural or stress-induced leaf senescence in many plant species (Ren et al., 2007; Morita et al., 2009; Zhang et al., 2011; Jespersen et al., 2016). In addition, leaf senescence has also been related to the up-regulation of senescence marker genes, such as *SAG12*, *h36*, and *l69* (Lee et al., 2001; Zhou et al., 2013). Dark stress leads to the overproduction of ROS in plants which causes chlorophyll degradation and leaf senescence (Rosenvasser et al., 2006). In this study, melatonin resulted in the down-regulation of expression of all six chlorophyll degradation-associated genes and three senescence marker genes in dark-treated leaves of perennial ryegrass. Our results indicate that melatonin may serve as a negative regulator for chlorophyll degradation-associated and senescence marker genes, which in turn contributes to the maintenance of higher chlorophyll content and photochemical efficiency during the dark treatment.

Leaf senescence is also characterized by loss of membrane stability or integrity, which is typically assessed as increased electrolyte leakage (Peever and Higgins, 1989; Rolny et al., 2011). Membrane lipid peroxidation due to excessive production of ROS in plants exposed to stresses also contributes to leaf senescence (Finkel and Holbrook, 2000; Muller et al., 2007; Bian and Jiang, 2009). Our study found the production of O_2_^-^ and H_2_O_2_ increased along with increases in both EL and MDA content in detached leaves of perennial ryegrass exposed to the dark treatment. However, exogenous melatonin treatment significantly decreased the endogenous O_2_^-^ and H_2_O_2_ production during the dark treatment. These results suggest that melatonin may alleviate dark-induced leaf senescence associated with membrane damage by reducing the accumulation of O_2_^-^ and H_2_O_2_.

Previous studies suggested that melatonin may reduce the oxidative damage by regulating ROS scavenging systems. Wang et al. (2012) found that melatonin treatment delayed the dark- and drought-induced senescence of detached apple leaves, which was attributed to the activation of the antioxidant enzymes in the ascorbate-glutathione pathway; however, their study examined only enzymes in this pathway. However, the effects of melatonin on ROS scavenging system were not systematically studied. Results in our study demonstrated that melatonin has no significant effects or inhibited the activity and transcript levels of antioxidant enzymes (APX, MDHAR, DHAR, GPX, and GR) in the ascorbate-glutathione and the glutathione peroxidase pathways during dark-induced leaf senescence of perennial ryegrass. Furthermore, the endogenous non-enzymatic antioxidants levels, such as free AsA and DHA, were significantly decreased by melatonin treatment; the content of free GSH and GSSG was not affected by melatonin. In addition, melatonin treatment significantly increased the activity of SOD and CAT and the transcript levels of *LpCuZnSOD*, *LpCuZnSOD*, *LpCuZnSOD*, and *LpCAT*. Our results strongly suggest that melatonin may play roles in regulating the SOD-CAT antioxidant pathway in perennial ryegrass, contributing to the suppression of dark-induced leaf senescence in perennial grass species. However, melatonin does not play such role with regard to the apple. Different mechanisms may be invovled for melatonin regulation of dark-induced leaf senescence for different plant species differing in their sensitivity to melatoinin.

As introduced before, leaf senescence is typically characterized by the decline in chlorophyll content and protein degradation, which causing the decline in forage value and turf quality of perennial grasses in heavily shaded areas in landscape, sports fields, and meadowlands. Our results indicated that exogenous melatonin suppressed dark-induced leaf senescence of perennial ryegrass. Therefore, melatonin may function as a signaling molecular which suppress stress-induced decline in forage value and turf quality of perennial ryegrass.

**FIGURE 10:**
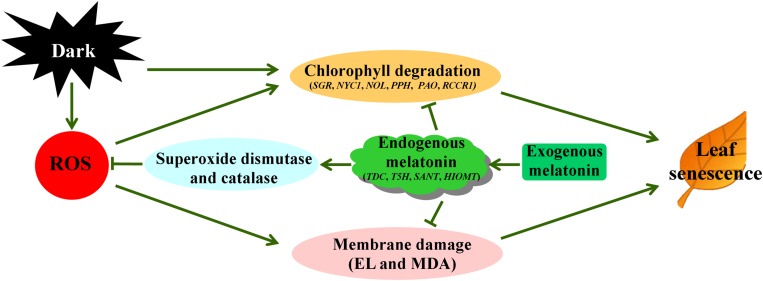
**Proposed pathways of melatonin regulation of dark-induced leaf senescence derived from results involving chlorophyll degradation and antioxidant metabolism**.

In summary, 20 μM melatonin treatment significantly delays dark-induced leaf senescence of perennial ryegrass. The exogenous application of melatonin effectively slowed the dark-induced yellowing or chlorophyll loss and decline of photochemical efficiency. The melatonin treatment also increased the cell membrane stability as manifested by lower electrolyte leakage and MDA content compared to water control. In addition, the transcription of chlorophyll degradation genes and senescence marker genes were also significantly suppressed, which strongly supports its beneficial role in regulation during darkness. Moreover, exogenous melatonin reduced the dark-induced oxidative stress damage by enhancing ROS scavenging toward regulating SOD and CAT pathway. Therefore, melatonin may respond differently to ROS scavenging pathways in different plant species, especially in dicots and monocots. Our results demonstrated that melatonin decreased dark-induced oxidant stress damage through regulating SOD-CAT ROS scavenging pathway in perennial ryegrass, as depicted in **Figure 10**. We hope that the beneficial effects of exogenous melatonin on leaf senescence offer new opportunities for its use in increasing the forage value and turf quality of perennial ryegrass.

## Author Contributions

JZ, HL, BX, and JL performed the experiment and analyzed the data. BH provided all financial support and design of this study. JZ and BH wrote and revised the manuscript. All authors read and approved the final manuscript. The authors declare no competing financial interests.

## Conflict of Interest Statement

The authors declare that the research was conducted in the absence of any commercial or financial relationships that could be construed as a potential conflict of interest.
